# Role of the Renin-Angiotensin-Aldosterone System beyond Blood Pressure Regulation: Molecular and Cellular Mechanisms Involved in End-Organ Damage during Arterial Hypertension

**DOI:** 10.3390/ijms17070797

**Published:** 2016-06-23

**Authors:** Natalia Muñoz-Durango, Cristóbal A. Fuentes, Andrés E. Castillo, Luis Martín González-Gómez, Andrea Vecchiola, Carlos E. Fardella, Alexis M. Kalergis

**Affiliations:** 1 Millenium Institute on Immunology and Immunotherapy, Departamento de Genética Molecular y Microbiología, Facultad de Ciencias Biológicas, Pontificia Universidad Católica de Chile, 8330025 Santiago, Chile; nmunoz4@uc.cl; 2 Millenium Institute on Immunology and Immunotherapy, Departamento de Endocrinología, Escuela de Medicina, Pontificia Universidad Católica de Chile, 8330074 Santiago, Chile; cristobal.fuentes.z@gmail.com (C.A.F.); acastilloram@gmail.com (A.E.C.); luismartin.gonzalezgomez@gmail.com (L.M.G.-G.); andreavecchiola@gmail.com (A.V.)

**Keywords:** renin-angiotensin-aldosterone system (RAAS), angiotensin II, aldosterone, end-organ damage, fibrosis, hypertension, primary aldosteronism

## Abstract

Arterial hypertension is a common condition worldwide and an important predictor of several complicated diseases. Arterial hypertension can be triggered by many factors, including physiological, genetic, and lifestyle causes. Specifically, molecules of the renin-angiotensin-aldosterone system not only play important roles in the control of blood pressure, but they are also associated with the genesis of arterial hypertension, thus constituting a need for pharmacological interventions. Chronic high pressure generates mechanical damage along the vascular system, heart, and kidneys, which are the principal organs affected in this condition. In addition to mechanical stress, hypertension-induced oxidative stress, chronic inflammation, and the activation of reparative mechanisms lead to end-organ damage, mainly due to fibrosis. Clinical trials have demonstrated that renin-angiotensin-aldosterone system intervention in hypertensive patients lowers morbidity/mortality and inflammatory marker levels as compared to placebo patients, evidencing that this system controls more than blood pressure. This review emphasizes the detrimental effects that a renin-angiotensin-aldosterone system (RAAS) imbalance has on health considerations above and beyond high blood pressure, such as fibrotic end-organ damage.

## 1. Introduction

Arterial hypertension (AH) is a frequent condition, affecting approximately 26% of the general population worldwide. This incidence rate is expected to rise to 30% by 2025 due to changing epidemiological profiles [1]. Current AH treatment involves lifestyle changes and drug therapy for lowering blood pressure. However, the underlying causes of AH are not addressed, resulting in a number of adverse effects and high treatment costs for patients. Furthermore, AH is one of the most prevalent risk factors for predominant coronary and brain-vascular diseases, such as heart diseases and strokes [2,3].

In more than 90% of cases, AH has an unknown etiology and is consequently diagnosed as essential, or primary AH. The remaining 10% of cases are diagnosed as secondary AH characterized by autonomous aldosterone production, caused by renal, cardiovascular, neurological, and endocrine diseases [4,5,6,7].

The renin-angiotensin-aldosterone system (RAAS) is one of the most important hormonal mechanisms in controlling hemodynamic stability by regulating blood pressure, fluid volume, and sodium-potassium balance. For that reason, an alteration in any molecules that compose RAAS contributes to developing AH [8]. Renin is synthesized in the kidneys as an inactive form and released into circulation in response to low levels of intratubular sodium, hypotension in the afferent arterioles of renal glomerulus, and sympathetic activation. In the blood stream pro-renin is activated by proteolytic and nonproteolitic mechanisms to produce the active form [9]. Here the active renin catalyzes the cleavage of the glycoprotein angiotensinogen, generating angiotensin I (Ang I). Ang I is cleaved by the angiotensin-converting enzyme (ACE) to produce angiotensin II (Ang II), the main effector in the RAAS, while neutral endopeptidases (EP) cleave angiotensin I to produce angiotensin-(1-7), another active peptide of this system that typically opposes the effects of Ang II. Most of the known proliferative and profibrotic effects of Ang II are through the angiotensin type 1 receptor (AT1-R), but it can also bind to the Ang II type 2 receptor (AT2-R) thereby triggering opposite effects to those at the AT1-R [10]. Angiotensin-(1-7) can also be produced by the cleavage of Ang II by angiotensin-converting enzyme 2 (ACE2), thus reducing the concentration in favor of AngII, which promotes vasodilatation on cardiac and vascular tissues [11,12].

Aldosterone is another effector molecule of the RAAS, whose synthesis and secretion are stimulated by Ang II through the AT1-R in the adrenal cortex. Through specific actions on the distal nephron of the kidney, aldosterone promotes sodium reabsorption, water retention, and potassium and magnesium loss, thereby modulating extracellular space volume and blood pressure [13]. The genomic effects of aldosterone occur through binding to the mineralocorticoid receptor (MR), translocating to the nucleus, interacting with the DNA, and thus promoting the transcription of genes that regulate electrolyte and fluid balance [14]. The MR has high affinity for both aldosterone and 11β-glucocorticoids, therefore binding of aldosterone to MR in kidneys is favored by the inactivation of cortisol to cortisone by the action of the enzyme 11β-hydroxysteroid dehydrogenase type 2. On the other hand, the aldosterone non-genomic pathways are through the AT1-R, G-protein-coupled receptor [15], and epidermal growth factor receptors [16]. Some of the effectors of these receptors are the MAPK/ERK1-2/p38 signaling pathways, mediating vascular remodeling, inflammation, and fibrosis [17], also involved in cardiorenal and metabolic diseases [18,19]. Once aldosterone is produced and secreted in the epithelial cells from the renal tubule [20] or vascular smooth muscle cells (VSMCs) [21], it induces the expression of genes related to water absorption, such as epithelial 3 sodium channel (ENaC), sodium-potassium ATPase, and serum/glucocorticoid regulated kinase 1 (SGK1) [22,23]. The main goal of these processes is to maintain a normal arterial blood pressure range by controlling water and electrolyte homeostasis.

**Figure 1:**
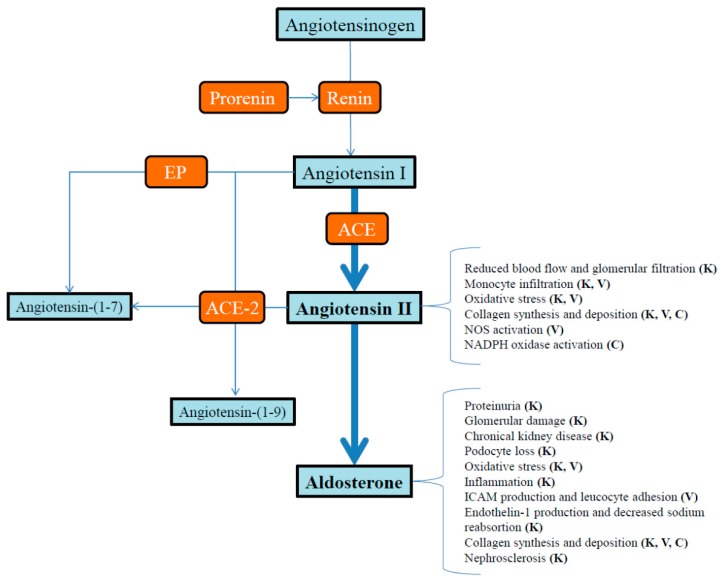
Detrimental effects of high levels of the RAAS molecules: angiotensin II and aldosterone during pathologic conditions such as, arterial hypertension and metabolic syndrome. **K**: Kidney; **V**: Vascular tissue; **C**: Cardiac tissue; **EP**: Endopedtidases; **ACE**: Angiotensin-converting enzyme; **ACE-2**: Angiotensin-converting enzyme 2.

Since blood pressure is finely tuned by the RAAS any unbalance in this system will produce arterial blood pressure alterations (Figure 1). Nowadays, the use of MR antagonism, ACE inhibitors, and AT-R antagonism as drugs used to control the blood pressure has also demonstrated additional benefits besides lowering blood pressure. In this review we present the available data regarding the role of RAAS molecules in generating end-organ damage during arterial hypertension.

## 2. The Role of Angiotensin II and Aldosterone-Dependent End-Organ Damage during Arterial Hypertension

The randomized Aldactone evaluation study (RALES) and Eplerenone post-acute myocardial infarction heart and survival study (EPHESUS) are clinical trials that evaluated the role of MR antagonism in patients with severe heart failure and patients with acute myocardial infarction complicated by left ventricular dysfunction, respectively. These trials demonstrated substantially reduced risks of morbidity and mortality when patients were treated by standard therapy together with MR antagonist spironolactone or eplerenone [24,25]. Further analysis of the EPHESUS cohort demonstrated that these positive results, which went beyond diuretic effects and potassium sparing, could be related to an unknown mechanism [26].

Also, in the prospective intervention trial captopril prevention project (CAPPP) [27] and Swedish Trial in Old Patients with Hypertension-2 study (STOP-Hypertension 2) [28] the potential benefits of ACE inhibitor (captopril) in addition to a conventional antihypertensive regimen of diuretics or β-blockers was evaluated in essential hypertension patients. Unfortunately, none of these studies showed significant differences in the outcome measured as cardiovascular morbidity and mortality, concluding that available drugs are just equally efficient at controlling AH. However, a meta-analysis study that encompasses clinical studies directed to evaluate the role of ACE inhibitors in cardiac hypertrophy, demonstrated that ACE drugs were more potent than β-blockers and diuretics reducing left ventricular mass index [29]. Additionally, clinical evidence suggests the renoprotective effects of ACE and AT-R inhibitors in hypertensive and diabetic patients with kidney failure [30], thereby designating these drugs as the first-line candidates to reduce the risk associated with left ventricular hypertrophy and kidney protection. Recently a study made in an animal model revealed that AT1-R expressed exclusively in the kidney is sufficient to generate hypertension mediated for angiotensin II and 2% NaCl infusion [31]. In addition, the authors demonstrated that cardiac hypertrophy and tissue damage in the heart is addressed by AT1-R expression in kidneys, rather than the expression of this receptor systemically. All of these findings suggest that deregulation of Ang II not only contributes to blood pressure regulation, but also to cardiovascular complications.

In primary aldosteronism (PA), aldosterone biosynthesis is independent of the RAAS regulation [32]. The prevalence of PA among hypertensive patients is between 6% and 10%, and this prevalence is greater in severely hypertensive patients [33,34]. Similarly, approximately 15% of essential hypertensive patients have inappropriate aldosterone levels [35,36,37]. The main subtypes of PA are bilateral adrenal hyperplasia (BAH), aldosterone-producing adenomas (APA), and idiopathic aldosteronism. APA patients present small, benign, and well delimited aldosterone-producing adenomas in the adrenal epithelium [38]. BAH patients present a hyperplasic zona glomerulosa of the adrenal cortex. Further, unilateral primary adrenal hyperplasia patients have unilateral aldosterone secretion without a detectable adenoma [39]. Other causes of PA are suprarenal carcinoma and several types of familial hyperaldosteronism (FH). Briefly, FH Type I (FH-I), also known as glucocorticoid-remediable aldosteronism, is produced by the expression of an adrenocorticotropic hormone-regulated aldosterone synthase, generated by an unequal crossover of the *CYP11B1* and *CYP11B2* genes [36,40,41,42]. FH-II is a glucocorticoid-resistant form of PA, present in patients with a familial history of PA caused by adrenal adenoma or hyperplasia. There is no etiopathogenic mechanism described for this type of FH [41,43,44]. FH-III is associated with several mutations in the G-protein activated Inward Rectifier Potassium Channel 4 coding gene *KCNJ5*, altering ionic homeostasis and causing increased aldosterone production from severely hyperplasic adrenal glands [45,46]. The recently described FH-IV is associated with mutations in calcium channels CACNA1D and CACNHA1H in patients who presented PA along with severe developmental abnormalities or juvenile hypertension and PA [47,48].

Patients with PA are more susceptible to cardiovascular complications, including left ventricular hypertrophy [49], stroke, non-fatal myocardial infarction, and atrial fibrillation [2]. Furthermore, an increased aldosterone-renin ratio in patients with resistant hypertension is considered a predictor of exacerbated cardiovascular injury, in addition to increasing the risk of developing uncontrolled resistant hypertension [5]. High plasma levels of aldosterone can also induce structural and functional alterations in the heart, kidneys, and blood vessels, such as vascular inflammation, myocardial fibrosis, nephrosclerosis, and tissue remodeling [33,49,50]. Moreover, PA patients have elevated levels of oxidative stress markers as compared to essential hypertensive patients. These markers include malondialdehyde, a lipid peroxidation marker of endothelial inflammation, and procollagen type 1 amino-terminal propeptide, a marker of myocardial collagen synthesis. The levels of these markers decrease after the specific treatment of PA [51].

The important role of RAAS molecules in hypertension has not only been demonstrated in the adult population, healthy children with hypertensive parents also have the tendency to present a higher aldosterone-renin ratio than healthy children with normotensive parents [52]. Additional evidence obtained from a group of hypertensive children demonstrated an association between the aldosterone-renin ratio and carotid intima–media thickness, a potential marker of hypertensive vascular damage [53]. These observations highlight the detrimental impact that RAAS molecules can have, even at an early age. This situation becomes even more relevant when considering the growing frequency of hypertension in children [52].

## 3. Fibrosis, Inflammation, and Their Relation to Arterial Hypertension

Fibrosis is defined as an overgrowth, hardening, and/or scarring of various tissues and is attributed to an excessive deposition of extracellular matrix components, including collagen [54]. Fibrosis is the result of chronic exposure to a wide range of stimuli, such as persistent infections, autoimmune reactions, allergic responses, chemical insults, radiation, and tissue injury. These stimuli induce the secretion of molecules involved in the activation of target cells related to the fibrotic process, including cytokines: IL-13, IL-21, transforming growth factor beta 1 (TGF-β1); chemokines: MCP-1, MIP-1β; angiogenic factors: VEGF; growth factors: PDGF; peroxisome proliferator-activated receptors; acute phase proteins; caspases; and components of the RAAS [54]. This process also plays an important role in the reparation/degradation ratio of the tissue repairing process. For example, if prolonged fibrosis occurs, molecules such as tissue inhibitors of metalloproteinases and metalloproteinases themselves are crucial.

The key cellular mediator of fibrosis is the myofibroblast [55]. Once activated, myofibroblasts serve as the primary collagen-producing cells. These cells are derived from a variety of sources, including resident mesenchymal cells, epithelial and endothelial cells in processes of epithelial/endothelial-mesenchymal transition, and circulating fibroblast-like cells originated from bone marrow stem cells. Also, the TGF-β1 signaling pathway is closely related with fibrosis development due to its capacity to induce the synthesis of extracellular matrix, to inhibit the effects of proteases that degrade extracellular matrix, and to increase the expression of cell surface integrins that interact with matrix components [56]. In adult bovine vascular endothelial cells, TGF-β1 induces its differentiation into smooth muscle cells. Previous authors have shown the disruption of cell—cell contacts and the acquisition of smooth muscle or myofibroblastic phenotype in these cells [57]. In addition, in vivo studies made in models of cardiac fibrosis corroborate that fibrosis is originated from endothelial cells through TGF-β1 stimulation, whereas the antagonist bone morphogenic protein 7 preserved the endothelial phenotype and reversed the endothelial to mesenchimal transition induced by TGF-β1 [58]. Finally, rat animal models of kidney fibrosis, established a close cross-talk between Ang II, aldosterone and TGF-β1 to generate abundant myofibroblasts recruitment at the sites of fibrosis [59]. The specific mechanisms involved in the fibrotic phenotype induced by Ang II and aldosterone will be discussed below.

The endothelial-mesenchymal transition induced by growth factors and cytokines is not a unique mechanism related with fibrosis. It was recently described that vascular adventitial fibroblasts have the ability to produce endotelin-1 in response to Ang II stimulation, which contributes to synthesis of extracellular matrix components, collagen and procollagen, suggesting a role during fibrosis development [60]. Additionally, vascular adventitial fibroblasts in response to Ang II induced the secretion of IL-6 and changing the expression of adhesion and migration of molecules P-selectin and ICAM-1 [61]. These changes impact in macrophage migration and accumulation in vascular tissue thereby linking the inflammation with a fibrotic phenotype.

In addition, cells of the immune system play pivotal roles in the genesis of fibrosis due to their role in recognizing and responding against danger associated molecular pattern molecules (DAMPs) via Toll-Like Receptors (TLRs). Specifically, during chronic hypertension, continuous cell damage and oxidative stress generate the release of many potential DAMPs from dead cells. Three of these molecules described as ligands of TLRs in arterial hypertensive models are: the high mobility group box 1 protein (HMGB1), mitochondrial DNA, and heparan sulfate [62,63,64].

In order to link inflammation, vascular remodeling and renal injury under hypertensive conditions are controlled by TLRs sensing, many experiments have been made in animal models. The first demonstration came from a TLR4 knockout mice model, which develops less-severe left ventricular hypertrophy following aortic banding compared to its respective sham controls [65]. Then, experiments made in mice infused with Ang II for two weeks, showed an up-regulation of mRNA-TLR4 in aortic segments, high expression of IL-6 and TNF-α, endothelial dysfunction, collagen deposition and vascular structural alterations, higher media thickness, and augmented media:lumen ratio compared to controls [66]. All of these markers were normalized after anti-TLR4 antibody treatment, but neither blood pressure nor left ventricular hypertrophy was modulated, indicating that inflammation through TLR4 signaling contributed only to pro-fibrotic phenotype. These results were also described in spontaneous hypertensive rats (SHR) treated with anti-TLR4 antibody [67]. Additionally, Wistar rats infused with aldosterone and 1% NaCl for four weeks augmented the cardiac and renal expression of TLR4 [68]. They links this augment with higher expression of cytokines (TNF-α, IL-1β and MCP-1), cardiac and renal collagen deposition, and fibrosis because a TLR4 signaling inhibitor, TAK-242, reversed these alterations. Importantly, Eissler et al. described that TLR4 expression in cardiac tissue during AH development is gradual during life in spontaneous hypertensive rats (SHR) compared with controls. The authors also demonstrated that antihypertensive drug therapy (Ramipril) avoided the overexpression of TLR4 and also the inflammatory response in cardiac tissue [64], directly indicating that chronic AH *per se* generates inflammatory changes in these tissues.

The uses of TLRs antagonism not only demonstrated their pivotal role in the fibrotic process in arterial hypertensive animal models, but also its participation in the perpetuation of organ damage. Meanwhile, the demonstration of endogenous ligand molecules as inflammatory mediators of AH and end-organ damage are still under research.

## 4. RAAS Molecules Implicated in Kidney Damage in Arterial Hypertension

Kidneys play a pivotal role in blood pressure control through several mechanisms, natriuresis and diuresis, neuro-hormonal factors such as RAAS, and the regulation of sympathetic nervous system activity. Kidneys are one of the organs affected during hypertension, resulting in functional and structural damage with consequent renal dysfunction, in turn inducing an exacerbated hypertension phenotype. Therefore, managing only blood pressure is insufficient to treat hypertension-associated end-organ damage [69].

Aldosterone can cause sustained renal damage in rat models of hyperaldosteronism, such as in deoxycorticosterone-high salt models (DOCA-salt) or through the chronic infusion of aldosterone in stroke-prone, spontaneously hypertensive rats drinking a 1% NaCl solution. Aldosterone-induced damage is characterized by proteinuria, collagen accumulation, and glomerular structural lesions [70,71]. These deleterious effects of aldosterone on kidney function appear to be due in part to the production of ROS [21]. Increased ROS production activates the mitogen-activated protein kinase (MAPK) pathway in renal cortical tissues, which in turn triggers renal injury [21]. In humans it has been reported in a study performed on eight patients with chronic kidney disease and persistent proteinuria treated with spironolactone, an antagonist of the AT1-R, in addition to ACE inhibitors therapy, a drastic reduction in proteinuria levels (54%) after four weeks of treatment [72]. Additionally, when type I and II diabetic patients with renal complications were treated with spironolactone, there was an important reduction in urinary albumin excretion and microalbuminuria. This observation suggests that spironolactone confers renal protection in diabetic individuals, but that other markers of endothelial dysfunction or of pro-inflammatory serum cytokines did not change [73,74]. Blocking the multitude of pro-fibrotic and pro-inflammatory effects of aldosterone could affect glomerular hemodynamics and could be beneficial in the long term by reducing progressive renal injury.

During hypertensive renal damage, the progressive impairment of renal function, or chronic kidney disease, is caused by the replacement of functional nephrons by fibrotic scar tissue, as triggered by hemodynamic and cellular factors [75]. Immediate consequences of this include the hypoperfusion of damaged nephrons, increased sodium retention, stimulation of RAAS, uremia, metabolic waste retention, and extensive proteinuria, among other effects [76]. Chronic kidney disease is characterized by interstitial macrophage infiltration, and these macrophages can synthetize and secrete several molecules related to fibrogenesis, such as fibroblast growth factors or cytokines (TGF-β, TNFα, IFN-γ), enzymes (e.g., ACE, plasminogen activators, collagenases) and their inhibitors (like tissue inhibitors of metalloproteinase (TIMPs)), matrix proteins (e.g., collagen, fibronectin, thrombospondin), and many other complement proteins, bioactive lipids, ROS, etc. [77]. Chronic kidney disease has a rapid progression and, generally, the patient dies before receiving a kidney transplant. Patients also suffer accelerated cardiovascular diseases, a condition known as cardiorenal syndrome. Cardiorenal syndrome can be induced by hypertension, inflammation, oxidative stress, and vascular calcification, among other conditions [78].

Several of the molecules related to fibrogenesis are being studied as potential targets for therapies. Of these, TGF-β mediates the processes of proliferation, apoptosis, and collagen synthesis and is one of the most ubiquitous profibrotic factors. This factor has been widely studied in the amelioration of chronic kidney disease due to the action of its upstream regulator, the macrophage migration inhibitory factor [79,80]. A recently reported novel interaction of TGF-β through the NOTCH signaling pathway indicates that this factor can positively regulate the hepatocyte growth factor, which could have a reparative effect in chronic kidney disease [81].

Components of the RAAS also directly affect the progression of renal fibrosis. Ang II acts on vascular smooth muscle cells, causing the vasoconstriction of both afferent and efferent arterioles. Consequently, this can lead to the development of both glomerular capillary hypertension and reduced renal blood flow. Intrarenal Ang II levels increase the sensitivity of tubuloglomerular feedback, which leads to renal blood flow and glomerular filtration reduction [75]. As a proinflammatory agent, Ang II also has non-hemodynamic effects that can modulate the chemotaxis, proliferation, and differentiation of monocytes into macrophages in endothelial, renal tubular and vascular smooth muscle cells [82].

Aldosterone also may promote fibrosis and target-organ dysfunction in hypertensive or diabetic patients by different ways, such as, plasminogen activator inhibitor stimulation, TGF-β1 and ROS [83,84,85]. Furthermore, it has been reported that aldosterone promotes the loss of glomerular podocytes, decreasing the slit-pore membrane integrity which leads to proteinuria [86,87]. Also, aldosterone induces oxidative stress in tubular and intersticial renal tissue and inflammation, promoting the salt-induced tubuloglomerular injury as part of the rapid non-genomics effects of this hormone [83].

New drug designs are focused on cardiovascular protection and reducing alterations in renal homeostasis to prevent the negative effects of MR activity under high aldosterone levels. For example, the MR antagonist spironolactone, used to treat hyperaldosteronism, causes hyperkalemia and decreases renal function. Finerenone, a dihydropyridine MR antagonist used to treat cardiac disease, recently evidenced organ-protective effects, no negative impacts on renal condition, and reduced electrolyte disturbance as compared with traditional steroid-based MR antagonists.

## 5. Vascular System and Mycoardium Damage by RAAS

High aldosterone levels have also been associated with myocardial hypertrophy, ventricular remodeling, proarrhythmogenic effects, myocardial ischemia, reduced coronary blood flow, and cardiac fibrosis leading to a maladaptive remodeling in the heart [88,89,90]. Those generated conditions not only promote fibrosis but also induce cell death, inflammation, and increase oxidant signaling [91,92,93]. Lemarié et al. showed an aldosterone-stimulated activation of ERK1/2, JNK, and nuclear factor κB (NF-κB) in VSMCs, which was dependent on the AT2-R [94] pathways related to vascular remodeling and oxidative stress. Additionally, aldosterone can stimulate NADPH oxidase complex activity, thereby increasing oxidative stress in the aorta [95], macrophages [96,97], and endothelial cells [70].

In the aorta, aldosterone stimulates NOX2 (gp91phox) and p22phox expression through a MR-dependent mechanism, and aldosterone might also increase the expression of p47phox through the AT2-R and MR-related mechanisms [98]. Conversely, in NOX2-deficient mice the aldosterone-mediated activation of NF-κB is prevented [99]. Likewise, p47phox-deficient mice reduced aldosterone-induced ROS production in the heart. Aldosterone can promote the activation of ERK1/2 and increase the expression of TGF-β1 in mesangial cells in the kidney and in cardiomyocyte cells through a non-genomic pathway [91,100]. This cytokine also induced the expression of connective tissue growth factor in cardiac fibroblasts and cardiac myocytes with a concomitant increase of fibronectin, collagen, and plasminogen activator inhibitor-1 [101]. In agreement with this, aldosterone/salt treatment in rats increases myocardial collagen synthesis and content, fibrosis, and profibrotic factors, including connective tissue growth factor, TGF-β, plasminogen activator inhibitor-1, matrix metalloproteinase-2, and TNF-α [102,103,104,105].

Activation of the MR by aldosterone regulates the expression of several genes involved in vascular fibrosis, calcification, and inflammatory damage in human VSMCs [21,106]. Moreover, it was recently shown that VSMCs express the intercellular adhesion molecule-1 in response to aldosterone-induced MR signaling. This expression promotes leukocyte adhesion by specifically associating with the lymphocyte function-associated antigen-1 expressed on the leukocyte surface [107]. Another interesting adhesion molecule that also functions in aldosterone regulation is endothelin-1 [108]. Aldosterone induces gene transcription to increase endothelin-1 protein levels, which inhibits sodium reabsorption. This inhibition paradoxically opposes the aldosterone-induced increase of sodium reabsorption in the renal collecting duct and systemic blood pressure [109]. Therefore, aldosterone appears to not only regulate the expression of genes involved in its function, but also genes that participate in its regulation.

In respect to Ang II, a recent study using a model of cardiac remodeling in hypertension salt-insensitive, guanylyl cyclase-A knockout mice [110] showed that chronic treatment with the MR antagonist eplerenone reduced the amount of brain and atrial natriuretic peptide, hypertrophy markers, and cardiac fibrosis markers, including TGF-β, collagen I, and collagen III. This data suggests that this model would present an upregulation of the AT2-R pathway. This effect has not been observed in models of guanylyl cyclase-A and AT2-R double knockout, thus underscoring the pro-hypertrophic and profibrotic actions of angiotensin II. However, the involvement of MR in this mechanism is still unclear, although it is possible that a relationship exists between MR and the cardiac AT2-R, acting on a remodeling pathway independent of blood pressure regulation, because ANP can directly downregulates *CYP11B2* gene expression in cultured adrenal cortical cells and neonate cardiomyocytes [111,112].

Ang II is also able to enhance ROS production and induce heart hypertrophy. The molecular pathways involved in these deleterious effects include the Rho small G proteins (e.g., RhoA, Rac1) [113]. These proteins act as a molecular switch, interacting with downstream targets. Both Rho-α and Rho-β can be up-regulated by angiotensin II through the action of AT2-R, and these Rho-kinases have been linked to ROS promotion and vascular inflammation mediated by the direct activation of endothelial nitric oxide synthase in the vasculature [114]. In turn, Rac1 is associated with aldosterone time- and dose-dependent increases in superoxide generation, an effect abolished by eplerenone [97]. In cardiac myocytes [115], Rho-kinases phosphorylate cardiac troponin to prevent tension. On the other hand, the inhibition of Rho-kinases with Fasudil prevents the development of cardiac hypertrophy and diastolic heart failure [113].

Another mechanism related to Ang II in cardiomyocytes is NADPH oxidase activation, promoting the formation of ROS such as superoxide [116], which can reduce the bioavailability of nitric oxide. Endothelial nitric oxide synthase-derived nitric oxide can react with superoxide to form peroxynitrite (ONOO^−^). An increase in peroxynitrite leads to excessive oxidation and the depletion of reducing agents such as 6*R*-5,6,7,8-tetrahydrobiopterin, thereby generating oxygen reduction and affecting nitric oxide synthesis through endothelial nitric oxide synthase uncoupling. This would result in the conversion of nitric oxide synthase.

## 6. Conclusions and Perspectives

**Figure 2:**
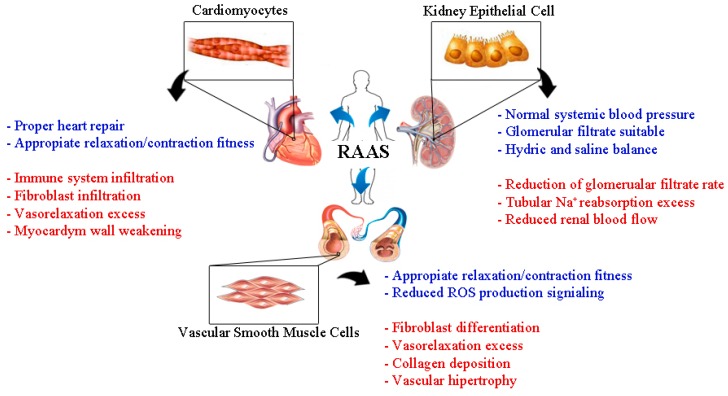
Physiological and detrimental roles of RAAS molecules in cardiac, vascular tissues and kidneys. Aldosterone and Ang II are the principal RAAS molecules involved in cardiovascular and renal system changes during hypertension. Both molecules are also involved in the physiological control of blood pressure (blue text), directly impacting cardiomyocytes, kidney epithelial cells, and vascular smooth muscle cells. During hypertension, excesses of these molecules have also been linked with cardiovascular and kidney tissue hypertrophy and fibrosis (red text).

This review puts forward the idea that RAAS is not only an important target in the control of blood pressure, but also is a key system in preserving organ structure and function (Figure 2). Due to these roles, there are continued efforts to develop new drugs against the components of RAAS, and, specifically, new MR antagonists are being developed to reduce the adverse effects of AH, such as electrolyte disturbances in complicated patients. For example, the use of finerenone a novel selective non-steroidal MR antagonist, in patients with diabetic nephropathy, demonstrated renoprotective effects after 90 days of treatment. Also, this study showed a dose-dependent reduction in the urinary albumin-creatinine ratio and the hyperkalemia, the worst side-effect of MR antagonists, was not observed in the treated group as compared with the placebo group [117,118]. Currently, a phase 2b clinical trial, named Mineralocorticoid Receptor antagonist Tolerability Study-Heart Failure (ARTS-HF), is being carried out to study the effects of finerenone in chronic heart failure patients with diabetes and/or chronic kidney disease [119]. Similar to the RALES and EPHESUS clinical trials, ARTS-HF includes patients concomitantly treated with ACE and angiotensin receptors blockers, reinforcing the need to effectively control RAAS in these patients.

As highlighted throughout this review, TGF-β is a key molecule in fibrosis. Here we focused in the induction of TGF-β expression and secretion stimulated by AngII and aldosterone stimulus. However, experiments made with pirfenidone, an antifibrotic drug, showed no effect in the inflammatory phenotype in the kidneys [120], thereby indicating a greater complexity of the fibrotic mechanisms in the control of end-organ damage in the context of alternated levels in RAAS.

In accordance with the notion of complex modulation of RAAS molecules, it has been described that extra-adrenal tissue, such as adipocytes, can express autonomously angiotensinogen, renin, AT1-R, and aldosterone synthase (CYP11β2) [121,122,123,124]. The local formation of Ang II appears to be increased in cases of obesity and leads to NFκB activation. This phnomena results in inflammatory cytokines secretions making the adipose tissue dysfunctional [125,126]. These effects can be blocked by AT1-R inhibition [127]. In addition to Ang II, adipocytes secrete mineralocorticoid-releasing factors that stimulate steroidogenesis in human adrenocortical cells, thereby explaining the higher aldosterone levels often observed in obese subjects. Cell culture studies demonstrate that fatty acid oxidation products, or endogenous ones from human adipocytes, could stimulate aldosterone synthesis [128], suggesting that free fatty acids could stimulate synthesis and release of aldosterone in adipocytes independently of angiotensin II. Interestingly, in humans, 12,13-epoxy-9-keto-10(trans)-octadecenoic acid (EKODE) plasma levels correlated with aldosterone plasma levels [128,129]. Instead, weight loss in humans results in decreased circulating angiotensinogen, plasma renin activity, and aldosterone concentrations [130,131,132]. In a similar way, in animal models of obesity, aldosterone increased inappropriately during high fat feeding despite a net positive sodium balance [133].

Based on the current growing problem of obesity and its link between adipose tissues in the control of RAAS; there exists an increased necessity to better understand this puzzle to face the new challenges in the AH population.

## Abbreviations

Ang II	Angiotensin II
ARTS-HF	Mineralocorticoid Receptor antagonist Tolerability Study-Heart Failure
AT1-R	Angiotensin II type 1 receptor
AT2-R	Angiotensin II type 2 receptor
EPHESUS	Eplerenone post-acute myocardial infarction heart and survival study
MR	Mineralocorticoid receptor
RAAS	Renin-Angiotensin-Aldosterone system
RALES	Randomized Aldactone evaluation study
ROS	Reactive oxygen species
